# DNA Methylation in Peripheral Blood: A Potential Biomarker for Cancer Molecular Epidemiology

**DOI:** 10.2188/jea.JE20120003

**Published:** 2012-09-05

**Authors:** Lian Li, Ji-Yeob Choi, Kyoung-Mu Lee, Hyuna Sung, Sue K. Park, Isao Oze, Kai-Feng Pan, Wei-Cheng You, Ying-Xuan Chen, Jing-Yuan Fang, Keitaro Matsuo, Woo Ho Kim, Yasuhito Yuasa, Daehee Kang

**Affiliations:** 1 Department of Preventive Medicine, Seoul National University College of Medicine, Seoul, Korea; 2 Cancer Research Institute, Seoul National University College of Medicine, Seoul, Korea; 3 Department of Epidemiology and Biostatistics, Tianjin Medical University Cancer hospital and Institute, Tianjin, China; 4 Department of Biomedical Sciences, Graduate School of Seoul National University, Seoul, Korea; 5 Department of Environmental Health, Korea National Open University, Seoul, Korea; 6 Division of Epidemiology and Prevention, Aichi Cancer Center, Nagoya, Japan; 7 Department of Cancer Epidemiology, Peking University School of Oncology, Beijing Cancer Hospital & Institute, Beijing, China; 8 Shanghai Institute of Digestive Disease, Renji Hospital, Shanghai Jiao-Tong University School of Medicine, Shanghai, China; 9 Department of Pathology, Seoul National University College of Medicine, Seoul, Korea; 10 Department of Molecular Oncology, Tokyo Medical and Dental University, Tokyo, Japan

**Keywords:** DNA methylation, blood-based biomarker, serum, plasma, leukocyte, peripheral blood

## Abstract

Aberrant DNA methylation is associated with cancer development and progression. There are several types of specimens from which DNA methylation pattern can be measured and evaluated as an indicator of disease status (from normal biological process to pathologic condition) and even of pharmacologic response to therapy. Blood-based specimens such as cell-free circulating nucleic acid and DNA extracted from leukocytes in peripheral blood may be a potential source of noninvasive cancer biomarkers. In this article, we describe the characteristics of blood-based DNA methylation from different biological sources, detection methods, and the factors affecting DNA methylation. We provide a comprehensive literature review of blood-based DNA methylation as a cancer biomarker and focus on the study of DNA methylation using peripheral blood leukocytes. Although DNA methylation patterns measured in peripheral blood have great potential to be useful and informative biomarkers of cancer risk and prognosis, large systematic and unbiased prospective studies that consider biological plausibility and data analysis issues will be needed in order to develop a clinically feasible blood-based assay.

## 1. INTRODUCTION

Biomarkers are biological molecules in body fluids or tissues that are quantitatively measured and evaluated as indicators of normal biological processes, pathogenesis, or pharmacologic response to a therapeutic intervention.^1^ Numerous types of biomarkers have been developed and used for early detection of cancer and prediction of prognosis and treatment response in cancer patients.

DNA methylation is a main component of the epigenetic mechanism that regulates embryonic development, transcription, chromatin structure, X-chromosome inactivation, genomic imprinting, and chromosome stability.^2^ DNA methylation occurs at the 5-carbon position of cytosine residues located in dinucleotide CpG sites. Although CpG within intergenic and transposable elements throughout the whole genome are mostly methylated, most CpG islands at the promoter region are unmethylated.^3^ In cancer, global loss of DNA methylation (global hypomethylation), as well as hypermethylation and hypomethylation of specific loci, has been observed. It has been suggested that altered DNA methylation initiates carcinogenesis and promotes cancer progression by activating oncogenes, suppressing tumor suppressor genes, and inducing chromosome instabilities.^4^

Because DNA is much more stable than other biological materials, such as RNA or protein, DNA methylation is easy to detect in small specimens and thus may be suitable for large-scale epidemiologic studies. Previous studies of the potential of DNA methylation as a cancer biomarker mainly used tumor tissue. However, an increasing number of studies are using body fluids such as urine, bronchial lavage fluid, breast milk, sputum, plasma and serum, and peripheral blood.^5^

In this review, we consider only studies that analyzed cell-free circulating DNA in plasma or serum or DNA from peripheral blood leukocytes. We describe the characteristics of blood-based DNA methylation from different biological sources, detection methods, and factors affecting DNA methylation. In addition, we comprehensively review the literature to investigate blood-based DNA methylation as a cancer biomarker, with a focus on studies using peripheral blood leukocytes.

## 2. ISSUES IN THE DEVELOPMENT OF METHYLATION-BASED BIOMARKERS USING PERIPHERAL BLOOD

### 2.1 Characteristics of different sources of blood-based DNA methylation

Blood-based DNA methylation is mainly derived from cell-free nucleic acid released from circulating cells in serum or plasma^6^ or DNA extracted from peripheral blood leukocytes or whole blood cells. Although cell-free DNA from circulating cells can serve as a surrogate for DNA from target tumor tissue, it is mixed with DNA from normal cells, which results in low specificity. In addition, the amount of DNA from serum or plasma is somewhat limited for use as a biomarker.^7^ However, a pooling method that uses DNA from groups of individuals has shown promise in identifying significant methylation markers.^8^

In contrast, the amount and quality of DNA extracted from peripheral blood leukocytes or whole blood are not usually a concern. Furthermore, it is common practice in many biospecimen repositories to bank DNA extracted from blood leukocytes. Although DNA from peripheral leukocytes is readily obtainable and easy to handle in laboratory processing and clinical use, the biological plausibility of DNA methylation in peripheral blood leukocytes and whole blood is uncertain.

Global methylation in peripheral blood leukocytes significantly differed between healthy controls and patients with pancreatic cancer, breast cancer, bilateral breast cancer, bladder cancer, and colorectal adenoma.^9^^–^^13^ Although methylation at specific loci in leukocytes was also observed in people with colorectal tumors, the correlation with target tissue showed little evidence of the origin of leukocyte methylation.^14^ Similarly ambiguous results were reported with regard to the correlation between methylation at specific loci in peripheral blood leukocytes/whole blood DNA and lung tissue DNA.^15^ Thus, it is controversial as to whether DNA methylation from peripheral blood leukocytes reflects methylation of target tissue.

It has been suggested that immunologic processes related to inflammation in cancer development lead to changes in leukocyte subpopulations, which could alter the epigenetic signatures in DNA from peripheral blood.^16^ Another possible explanation is that epigenetic change due to methylation is associated with the genetic variants of specific cancers.^16^^,^^17^ Increased knowledge of the origin and nature of DNA methylation in peripheral blood leukocytes is needed to determine whether DNA methylation in such cells can serve as an informative biomarker. In addition, future studies should investigate variation in DNA methylation in heterogeneous leukocyte subpopulations and differences in the processing of white blood cells.

### 2.2 Detection methods

To identify a sensitive and specific biomarker, it is important to select an appropriate method that is standardized, robust, sensitive, and cross-validated between laboratories and across different platforms. The details of such methods have been comprehensively reviewed in several articles.^4^^,^^18^^–^^21^ We will describe the advantages and disadvantages of extant methods of DNA methylation and will focus on the methods frequently used to detect DNA methylation in peripheral blood. Table 1 summarizes the methods used to assess DNA methylation and several of the important factors to be considered in method selection, such as analytical sensitivity measured as limit of detection (LOD), quantitativeness, and time required.

**Table 1.** Comparison of selected characteristics related to laboratory validation of various DNA methylation assays

Technology	LOD^a^	Quantitativeness	Time requirement	Reference
***Candidate gene***^b^				
MSP	0.1	No	<2 hrs/96	74
Bisulfite sequencing	>2	Yes	>4 hrs/96	75
Pyrosequencing	2	Yes	4 hrs/96	76
COBRA	3	No/Yes	5 hrs/80–160	77, 78
MS-SnuPE	0.1	Yes	5 hrs/80–160	79, 80
MethyLight	0.01	Yes	<2 hrs/96	81
MS-FLAG	0.01	Yes	<2 hrs/96	82
***Genome-wide profiling***				
RLGS	—	No	5–14 d	83, 84
MSRF	—	No	<5–14 d	85
MeDIP/MIRA	0.1	Yes	2–3 d/12	86
Beadchip (Infinium)	2.5	Yes	3 d/96	22
***5-methylcytosine contents***				
HPLC	>1 uM	Yes	15–60 min	23
HPCE	1 uM	Yes	10 min	87
LC-ESI-MS	0.2 fmol	Yes	15 min	24

Abbreviations: LOD, limit of detection; MSP, methylation-specific PCR; COBRA, combined bisulfite restriction analysis; MS-SnuPE, methylation-sensitive single-nucleotide primer extension; MS-FLAG, methylation-specific fluorescent amplicon generation; RLGS, restriction landmark genomic scanning; MeDIP, methyl-DNA immunoprecipitation; MIRA, methylated-CpG island recovery assay; HPLC, high-performance liquid chromatography; HPCE, high-performance capillary electrophoresis; LC-ESI-MS, liquid chromatography–electrospray ionization-mass spectrometry.

^a^Ratio of methylated cytosine to unmethylated cytosine (for the gene-specific methylation approach [%]) or the amount of DNA (for the global DNA methylation approach).

^b^PCR amplification of desired target was conducted after bisulfite conversion.

Methylation at specific loci can be examined in selected candidate genes or genome-wide. Most candidate gene analyses are based on bisulfite treatment and PCR/sequencing followed by quantitative measurement of DNA methylation level. The process of bisulfite treatment and PCR can be done using a relatively small amount of low-quality DNA and is thus suitable for DNA derived from serum or plasma, as well as that from peripheral blood leukocytes. There are 3 types of methods to measure methylation level at specific loci, ie, real-time PCR-based methods (methylation-specific melting curve analysis [MS-MCA], methylation-sensitive high-resolution melting [MS-HRM], HeavyMethyl, MethyLight, melting curve methylation-specific PCR [McMSP], sensitive melting analysis after real-time methylation-specific PCR [SMART-MSP], methylation-specific fluorescent amplicon generation [MS-FLAG], and quantitative analysis of methylated alleles [QAMA]); sequencing-based methods (direct bisulfite sequencing and pyrosequencing); and gel electrophoresis-based methods (combined bisulfite restriction analysis [COBRA] and methylation-sensitive single-nucleotide primer extension [MS-SnuPE]).

Methyl-sensitive enzyme digestion, affinity enrichment, and the bisulfite treatment-based array are used for genome-wide profiling. There are a variety of enzyme digestion methods such as restriction landmark genomic scanning (RLGS), methylation-sensitive restriction fingerprinting (MSRF), differential methylation hybridization (DMH), and methylated CpG island amplification/representational difference analysis (MCA-RDA). However, enzyme digestion methods generally require a large amount of DNA (approximately 10 µg) and have limited coverage for DNA as compared with affinity enrichment methods (methylated-CpG island recovery assay [MIRA], methyl-DNA immunoprecipitation [MeDIP], tiling array, CpG island microarray, and next-generation sequencing [NGS]) or a bisulfite treatment-based array.^20^ Neither enzyme digestion methods nor affinity enrichment methods can focus on specific CpG sites of interest, and thus the results are likely to be biased toward CpG-dense regions, due to the different efficiency of enzyme digestion and changes in antibody combination through different runs. In contrast, the bisulfite treatment-based array combined with bead array technology, such as the Infinium methylation array, requires only a small amount of DNA (250–500 ng) and is highly reproducible (*r^2^* > 0.998).^19^ The correlation coefficients of the Infinium and GoldenGate assays, pyrosequencing, and bisulfite sequencing were reported to be greater than 0.8.^22^ Methods for genome-wide profiling can be classified as array-based analysis and deep sequencing, according to genotyping technology. Bock et al^21^ compared the different platforms of 4 types of genome-wide DNA methylation-mapping technologies, including methylated DNA immunoprecipitation sequencing (MeDIP-seq), methylated DNA capture by affinity purification sequencing (MethylCap-seq), reduced representation bisulfite sequencing (RRBS), and the Infinium methylation assay. They reported that the accuracy of the RRBS and Infinium assays was slightly higher than that of the other 2 methods. However, the genomic coverage of MeDIP-seq and MethylCap-seq was higher than that of the RRBS and Infinium assays.

Global DNA methylation can be measured by direct and indirect quantification assays. Direct methods, such as the [^3^H]-methyl incorporation assay, high-performance liquid chromatography (HPLC), high-performance capillary electrophoresis (HPCE), and liquid chromatography–electrospray ionization tandem mass spectrometry (LC-ESI-MS/MS), measure 5-methylcytosine content throughout the genome. LC-based methods are the most common and have good reproducibility. LC-ESI-MS/MS needs less DNA than the other methods (1 µg for LC-ESI-MS/MS vs 5–10 µg for HPLC) and requires less time per sample (15–60 min for separation using HPLC vs <15 min for separation using LC-ESI-MS/MS).^23^^,^^24^ Direct measurement of 5-methycytosine content in DNA requires a large amount of DNA and is labor intensive, which led to the development of an indirect method that measures methylation levels of repetitive elements (*ALU*, *LINE1*, and *SAT*). The repetitive elements represent over 45% of CpG dinucleotides in the human genome and are correlated with 5-methylcytosine levels throughout the genome.^25^ Using MethyLight, Weisenberger et al reported that the methylation level of repeated elements was correlated with global DNA methylation as measured by HPLC.^25^ However, it is uncertain whether methylation levels of repetitive elements perfectly represent 5-methylcytosine content.

### 2.3 Factors affecting DNA methylation

A valid biomarker should have greater interindividual than intraindividual variation and higher interclass than intraclass correlation coefficients. Several studies have shown that DNA methylation pattern changed over time according to various endogenous and exogenous factors, such as demographic and lifestyle factors (age, race, sex, smoking, and alcohol consumption), diet intake (folate, vitamin B, green tea, and phytoestrogen), environmental exposures (arsenic, cadmium, and benzene), and disease status (infection and cancer).^26^

The methylation levels of several genes were shown to be correlated with smoking, alcohol consumption, and high fat intake, although most studies using cell-free DNA in serum and/or plasma did not show any significant association.^27^^–^^29^ Interestingly, several well-designed epidemiology studies found that obesity, dietary pattern, and physical activity were associated with global methylation in DNA extracted from peripheral blood leukocytes. Recently Teschendorff et al^30^ conducted a genome-wide scan of 27 000 CpG sites in 261 postmenopausal women and found that most CpGs were hypermethylated with age. Breitling et al^31^ used the same approach and found that specific methylation of *F2RL3* was associated with tobacco smoking in 177 individuals and validated this result using mass spectrometry and the Sequenom EpiTYPER in 316 individuals. In addition, a very recent review^32^ showed that demographic factors (age, sex, race, family history of cancer, education and race), environmental factors (benzene, organic pollutants, lead, arsenic, air pollution), behavioral factors (smoking, alcohol drinking, physical activity, and folate intake), and even genetic variation in carbon-metabolizing enzymes were associated with global methylation level in lymphocyte DNA.^9^^,^^33^^–^^38^ Thus, potential confounding factors affecting methylation status should be considered in the design of studies evaluating DNA methylation as a cancer biomarker. Furthermore, findings in the discovery stage should be cross-validated using independent samples.

## 3. PREVIOUS STUDIES OF DNA METHYLATION IN PERIPHERAL BLOOD AS A BIOMARKER OF CANCER RISK AND PROGNOSIS

We searched MEDLINE (PubMed) using the following keywords: DNA methylation, cell in serum and/or plasma, peripheral blood leukocytes, and cancer. We also searched the references of the retrieved articles. Among the identified studies, we included only those that had an epidemiologic design (cohort study, case-control study, or case-only study) and investigated DNA methylation as a biomarker of cancer risk and prognosis.

### 3.1 Circulating cell-free DNA methylation as a cancer biomarker

Table 2 shows the studies that used circulating cell-free DNA in serum and/or plasma to investigate DNA methylation as a diagnostic biomarker of cancer. In studies using circulating nucleic acid, the candidate gene approach was more frequent than genome-wide analysis, possibly due to the limited amount of available DNA. Previous studies have focused on genes and pathways related to carcinogenesis and tumor progression, namely, tumor oncogenes (*TMEFF2*, *HPP1*, and *PGR*), tumor suppressor genes (*TIG1*, *APC*, *RASSF1A*, and *DAPK*), cell cycle-related genes (*P16^INK4^*, *14-3-3δ*, *GSTP1*, *p15*, *p16*, *RAR-β*, and *SEPT9*), cell adhesion molecules (*CDH1* and *CDH13*), cell proliferation-related genes (*ESR1*, *MYOD*, and *PTGS2*), tissue invasion- and metastasis-related genes (*TIMP3* and *E-cadherin*), and others (*hMLH1*, *NGFR*, *AR*, *MGMT*, *HLTF*, and *TPEE*). As compared with healthy individuals, DNA methylation of the tumor suppressor genes *APC* and *RASSF1A* was altered in circulating cell-free DNA of patients with breast or gastric cancer.^39^^–^^42^ DNA methylation of cell cycle-related genes such as *P16^INK4^*, *14-3-3δ*, *GSTP1*, *p15*, *p16*, *RAR-β*, and *SEPT9* was reported in bladder, lung, prostate, and colorectal cancer and in head and neck squamous cell carcinoma.^43^^–^^48^ However, almost all these studies failed to adjust for confounding factors, including well-known cancer risk factors. Only 1 study showing promoter hypermethylation in *MGMT*, *P16^INK4α^*, *RASSF1A*, *DAPK*, and *RAR-β* in lung cancer patients reported risk estimates adjusted for age, sex, smoking status, and protein tumor marker.^47^ Recently, Epigenomics AG^49^ conducted a multistage study to identify and validate methylation biomarkers for colorectal cancer. In the first stage, candidate markers were selected by restriction enzyme-based discovery methods using colorectal cancer tissue and normal tissue. In the second stage, candidate genes identified in the first stage (ie, *TMEFF2*, *NGFR*, and *SEPT9*) were confirmed by real-time assays using DNA from circulating plasma cells.^48^ Finally, *SEPT9* methylation identified in the second stage was validated in a clinical trial.

**Table 2.** Associations between serum and/or plasma DNA methylation and cancer risk

Genes	Sample size (cases/controls)	Assay	Source	Results^a^	Reference

				OR (95% CI), *P*	
**Breast cancer**					
*RASSF1A*	33/29	MSP	Plasma	Case: 12%; Control: 0%	40
*APC*	79/19	QMSP	Serum	*APC*: *P* = 0.03	39
*ESR1*				*ESR1*: *P* = 0.33	
*RASSF1A*				*RASSF1A*: *P* = 0.002	
*APC*	36/30	EpiTyper assay	Plasma/Serum	*APC*: *P* < 0.001	88
*BIN1*				*BIN1*: *P* < 0.001	
*BMP6*				*BMP6*: *P* = 0.068	
*BRCA1*				*BRCA1*: *P* < 0.001	
*CST6*				*CST6*: *P* < 0.002	
*ESR-b*				*ESR-b*: *P* = 0.122	
*GSTP1*				*GSTP1*: *P* = 0.003	
*P16*				*P16*: *P* < 0.001	
*P21*				*P21*: *P* < 0.0001	
*TIMP3*				*TIMP3*: *P* < 0.0001	
**Bladder cancer**					
*P16^INK4α^*	86/49	MSP	Serum	13.6 (1.8–105.2), *P* = 0.0009	43
**Gastric cancer**					
*RASSF1A*	47/30	MSP	Serum	*P* < 0.01	41
*APC*	60/22	MethyLight	Serum	*APC*: *P* = 0.08	56
*hMLH1*				*hMLH1*: *P* = 0.03	
*TIMP3*				*TIMP3*: *P* = 0.005	
**Head and neck squamous cell carcinoma**					
*p15*	20/24	MethyLight	Plasma	*p15*: *P* = 0.0037	46
*p16*				*p16*: *P* = 0.016	
**Lung cancer**					
*DAPK*	100/100	MSP	Serum	At least 1 gene positive: 5.3 (2.4–11.7) At least 2 genes positive: 5.9 (1.5–22.7)	47
*MGMT*					
*P16^INK4α^*					
*RASSF1A*					
*RAR-β*					
**Nasopharyngeal carcinoma**					
*CDH1*	41/43	QMSP	Plasma	*CDH1*: *P* < 0.0001	89
*DAPK*				*DAPK*: *P* = 0.002	
*p15*				*p15*: *P* = 0.002	
*p16*				*p16*: *P* < 0.0001	
*RASSF1A*				*RASSF1A*: *P* = 0.235	
				At least 1 gene positive: *P* < 0.001	
**Prostate cancer**					
*GSTP1*	168/11	QMSP	Serum	*GSTP1*: *P* < 0.0001	45
*PTGS2*				*PTGS2*: *P* = 0.05	
*TIG1*				*TIG1*: *P* = 0.038	
*14-3-3δ*	46/49	MSP	Serum	*14-3-3δ*: *P* = 0.03	44
*AR*				*AR*: *P* > 0.05	
*GSTPI*				*GSTP1*: *P* < 0.001	

Abbreviations: MSP, methylation-specific PCR; QMSP, quantitative methylation-specific PCR; MSRE, methylation-sensitive restriction enzyme; HPCE, high-performance capillary electrophoresis; LC/MS, liquid chromatography/mass spectrometry; COBRA, combined bisulfite restriction analysis.

^a^Hypermethylation of genes increased cancer risk.

Several studies have evaluated the potential of DNA methylation as a prognostic biomarker of cancer. Table 3 summarizes studies that investigated DNA methylation in circulating cell-free DNA as a prognostic biomarker. In a variety of cancers, including hepatocellular carcinoma, breast, bladder, cervical, and colorectal cancer,^50^^–^^54^ both the methylation pattern in specific loci and global methylation level were observed with regard to disease-free survival and/or overall survival. Altered DNA methylation of *APC* was commonly associated with overall survival in breast, gastric, and esophageal cancer.^28^^,^^55^^–^^57^ Promoter methylation of cell adhesion molecule genes *CDH1* and *CDH13* and cell proliferation-related gene *MYOD* was associated with relapse-free survival in cervical cancer.^53^^,^^58^ Promoter methylation of *GSTP1* was associated with disease-free survival in prostate cancer, and *hMLH1* was associated with overall survival in ovarian cancer.^59^^,^^60^ In hepatocellular carcinoma, global hypomethylation quantified with *LINE1* was associated with overall survival.^29^ A few studies shown in Table 4 evaluated the predictive values, including sensitivity and specificity, of DNA methylation in predicting cancer outcomes. In colorectal cancer and prostate cancer, multimarker analysis had much higher sensitivity and specificity than did single-marker analysis.^61^^,^^62^ Moreover, in some cases, the sensitivity and specificity of methylation markers were reported to be moderately higher than those of present diagnostic markers in clinical use, such as PSA for prostate cancer, fecal occult blood testing for colorectal cancer, CA125 for ovarian cancer, and combined analysis of CA19-9 and CA125 for pancreatic cancer.

**Table 3.** Associations between serum and/or plasma DNA methylation and cancer prognosis

Genes	Sample size (events/non-events)	Assay	Results^a^		Reference

			Outcome	HR (95% CI), *P*	
**Breast cancer**					
*RASSF1A*^b^	13/148	MethyLight	Relapse-free survival	5.1 (1.3–19.8)	55
*APC*	17/85	MethyLight	Overall survival^c^	*APC/RASSF1A*: 5.7 (1.9–16.9), *P* = 0.002	28
*RASSF1A*					
*PITX2*	428	MethyLight	Overall survival Distant disease-free survival	*PITX2*: 3.4 (1.2–9.8), *P* = 0.021	51
*RASSF1A*				*RASSF1A*: 5.6 (2.1–14.5), *P* < 0.001	
				*RASSF1A*: 3.4(1.6–7.3), *P* = 0.002	
**Bladder cancer**					
*P^14ARF^*	12/15	MSP	Relapse-free survival	*P* = 0.03	52
**Cervical cancer**					
*MYOD1*	53/40	MethyLight	Relapse-free survival	*P* = 0.04	58
*CDH1*	53/40	MethyLight	Relapse-free survival	*CDH1/CDH13*: 2.5 (1.3–4.6), *P* = 0.005	53
*CDH13*					
**Colorectal cancer**					
*HLTF*	28/77	MethyLight	Overall survival	*HLTF*: 3.0 (1.4–6.4), *P* = 0.008	54
*HPP1*				*HPP1*: 5.1 (2.2–11.6), *P* = 0.001	
*hMLH1*				*hMLH1*: 1.4 (0.6–3.1), *P* = 0.425	
				*HLTF/HPP1*: 3.4 (1.4–8.1), *P* = 0.007	
**Esophageal cancer**					
*DAPK*	59	QMSP	Overall survival	0.2 (0.0–0.5), *P* = 0.0036	90
*APC*	52	QMSP	Overall survival^c^	*P* = 0.016	57
**Gastric cancer**					
*APC*	32/26	MethyLight	Overall survival	*APC*: *P* = 0.006	56
*CDH1*				*CDH1*: *P* = 0.006	
**Hepatocellular carcinoma**					
*LINE1*	85	COBRA	Overall survival	1.7 (1.1–2.8), *P* = 0.021	29
**Lung cancer**					
*DAPK*	76	QMSP	Overall survival	*DAPK*: *P* = 0.587	90
*MGMT*				*MGMT*: *P* = 0.202	
*14-3-3δ*	75/40	MSP	Overall survival	2.1 (1.2–3.5), *P* = 0.006	91
**Ovarian cancer**					
*hMLH1*^b^	78/53	MSP	Overall survival	2.0 (1.2–3.3), *P* = 0.007	59
**Prostate cancer**					
*GSTP1*	55/55	REQP	Disease-free survival	4.4 (2.2–8.8), *P* < 0.001	60

Abbreviations: MSP, methylation-specific PCR; QMSP, quantitative methylation-specific PCR; MSP, methylation-specific PCR; COBRA, combined bisulfite restriction analysis; REQP, restriction endonuclease quantitative PCR.

^a^Hypermethylation of genes worsened prognosis.

^b^Measurements were done at disease endpoint.

**Table 4.** Population validation of methylation-based biomarkers using plasma/serum DNA

Genes	Sample size (cases/controls)	Assay	Source	Sensitivity (%)	Specificity (%)	Reference
**Breast cancer**						
*APC*, *GSTP1*, *RASSF1A*, *RARβ2*	93/76	QMSP	Plasma	62	87	92
**Colorectal cancer**						
*APC*, *MGMT*, *RASSF2A*, *Wif-1*	243/276	MSP	Plasma	87	92	62
*SEPT9*	97/172	Real-time qPCR	Plasma	72	93	^93^
**Hepatocellular carcinoma**						
*P15*, *P16*, *RASSF1A*	50/50	MSP	Serum	84	94	50
**Ovarian cancer**						
*BRCA1*, *HIC1*, *PAX5*, *PGR*, *THBS1*	33/33	MethDet test	Plasma	85	61	94
**Pancreatic cancer**						
*CCND2*, *PLAU*, *SOCS1*, *THBS*, *VHL*	30/30	MethDet test	Plasma	76	59	94
**Prostate cancer**						
*GSTP1*, *RASSF1*, *RARB2*	83/40	MSP	Serum	89	—	61

Abbreviations: QMSP, quantitative methylation-specific PCR; MSP, methylation-specific PCR.

### 3.2 Leukocyte DNA methylation as a cancer biomarker

Table 5 shows the associations between methylation patterns of DNA extracted from leukocytes and cancer risk. Methylation at specific loci in DNA from peripheral blood leukocytes/whole blood was first reported in lung cancer.^15^ In a nested case-control study (*n* = 100), the researchers hypothesized that methylation status in DNA extracted from whole blood would reflect the status of lung tissue DNA. They identified a correlation between *p53* gene hypomethylation in whole blood DNA and lung cancer. In breast cancer, methylation of specific loci in *ERT* (*NUP155* and *ZNF217*), *PCGT* (*TITF1*, *NEUROD1*, and *SFRP1*), and *DMHR* (*PTGS2*) of DNA from extracted peripheral blood cells was associated with breast cancer risk.^13^ Flanagan et al^10^ compared the methylation pattern of peripheral blood DNA using a custom methylation microarray analysis covering 4 Mb with 51 candidate genes from 14 bilateral breast cancer cases and 14 normal controls and validated their initial findings regarding the tiled region around *ATM* in 190 pairs of cases and controls. The results proved their hypothesis that some systemic epigenetic changes would be detected in peripheral blood DNA in breast cancer. However, in a case-control study using lymphocyte DNA from 97 colon cancer cases and 190 age- and sex-matched controls, the mean fraction of CpG methylation was identical among cases and controls, and there was no relationship between colon cancer risk and quartile levels of CpG methylation.^63^

**Table 5.** Associations between DNA methylation in peripheral blood leukocytes and cancer risk

Disease/Genes	Sample size (cases/controls)	Assay	Results	Reference

			OR (95%CI)	
**Breast cancer**				
*NEUROD1*	353/730	MethyLight	1.5 (1.1–2.0)	13
*NUP155*			1.4 (1.0–1.9)	
*SFRP1*			1.4 (1.1–1.9)	
*TITF1*			1.5 (1.1–2.2)	
*ZNF217*			1.5 (1.1–2.0)	
*ATM*	190 /190	MSRE-microarray, Pyrosequencing	High vs Low: 3.2 (1.8–5.9)	10
5-mdC	176/173	LC/MS	Middle vs High: 1.5 (0.8–2.7) Low vs High: 2.9 (1.7–4.9)	9
*LINE1*	40/40	MethyLight	*P* > 0.05	95
*ALU*			*P* > 0.05	
*SAT*			*P* = 0.01	
**Bladder cancer**				
mC contents^a^	775/397	HPCE	Q1 vs Q4: 2.7 (1.8–4.0) Q2 vs Q4: 1.6 (1.1–2.4) Q3 vs Q4: 2.1 (1.4–3.1)	11
*LINE1*	510/528	Pyrosequencing	Middle vs High: 1.3 (0.8–2.3) Low vs High: 1.9 (1.2–3.1)	64
Gene panels	111/119	Illumina Infinium beadchip array	AUC: 0.8 (0.7–0.8)	64
(9 CpG sites)				
*LINE1*	285/465	Pyrosequencing	Low vs High: 1.8 (1.1–2.9)	96
**Colon cancer**				
*IGFII*	97/190	SOMA assay	Q1 vs Q4: 1.1 (0.5–2.4) Q2 vs Q4: 1.2 (0.5–2.6) Q3 vs Q4: 1.4 (0.6–3.0)	63
**Colorectal adenoma**				
mC contents	115/115	LC/MS	Middle vs Low: 0.7 (0.3–1.5) High vs Low: 0.2 (0.1–0.5)	12
**Gastric cancer**				
*ALU* *LINE1*	302/421	Pyrosequencing	Low vs High: 1.3 (0.9–1.9) Low vs High: 1.4 (0.9–2.0)	67
**Hereditary diffuse gastric cancer**				
*CDH1*	22/21	Pyrosequencing	25% of cases displayed high *CDH1* allelic expression imbalance	97
**Head and neck squamous cell carcinoma**				
*LRE1*	278/526	COBRA	Middle vs High: 1.3 (0.9–2.0) Low vs High: 1.6 (1.1–2.4)	34
**Lung cancer**				
*P53*	100/100	*HpaII* quantitative PCR assay	2.2 (1.0–4.7)	15
*CSF3R*	138/138	Illumina beadchip assay, pyrosequencing	3.9 (2.0–6.1)	65
*ERCC1*			1.5 (1.1–2.0)	
**Ovarian cancer**				
Gene panels	255/148	Illumina Infinium	AUC: 0.8 (0.7–0.9)	16
(100 CpG sites)		beadchip array		
**Pancreatic cancer**				
*IL10*	220/220	Illumina VeraCode array	AUC: 0.8	17
*LCN2*				
*ZAP70*				
*AIM2*				
*TAL1*				

Abbreviations: MSRE, methylation-sensitive restriction enzyme; HPCE, high-performance capillary electrophoresis; 5-mdC, 5-methyldeoxycytosine; LC/MS, liquid chromatography/mass spectrometry; AUC, area under the curve; COBRA, combined bisulfite restriction analysis.

In genome-wide scanning using leukocyte DNA, potential methylation biomarkers were identified in several cancers, including ovarian, pancreatic, bladder, and non–small-cell lung cancers.^16^^,^^17^^,^^64^^,^^65^ Teschendorff et al^16^ evaluated the methylation signature of 27 000 CpG sites using DNA extracted from peripheral blood in 113 pretreatment ovarian cancer cases and 148 healthy controls and validated the results among an independent set of 122 post-treatment ovarian cancer cases. Marsit et al^64^ used an Infinium methylation chip and identified a panel of DNA methylation loci that might serve as a useful biomarker of bladder cancer. In the first phase, they identified a panel of 9 CpG loci in 112 cases and 118 controls and then validated the findings in 111 cases and 119 controls. In the discovery stage of another genome-wide scanning study, *CSF3R* and *ERCC1* gene methylation was identified as a biomarker of small-cell lung cancer using a methylation array of 1505 CpG sites in 39 small-cell lung cancer cases and 44 matched controls, which was validated in an independent set of 138 matched case-control pairs using pyrosequencing.^65^ Pedersen et al^17^ conducted a 2-phase study using the GoldenGate methylation Beadchip for phase I and the Illumina custom VeraCode methylation assay for phase II in 220 pairs of cases and controls. They found that a panel of genes (*IL10*, *LCN2*, *ZAP70*, *AIM2*, and *TAL1*) might be a diagnostic biomarker of pancreatic cancer.

In addition to methylation in specific genes, associations between global hypomethylation in peripheral blood leukocytes and cancer risk were reported for several cancers, including breast, bladder, colorectal adenoma, head and neck squamous cell carcinoma, tongue and esophageal cancer, and gastric cancer, as shown in Table 5.^9^^,^^11^^,^^12^^,^^34^^,^^64^^,^^66^^–^^68^ Some of these studies attempted to control for confounding factors in the association between methylation level and disease status by selecting subjects in certain cancer stages, as shown in a study of colorectal cancer,^12^ and by using stratified analysis of confounding factors, such as smoking status in a study of bladder cancer.^11^ Only 1 study investigated DNA methylation in peripheral blood leukocytes as a prognostic marker of cancer. Using pyrosequencing, Al-Moundhri et al found that global methylation and promoter methylation in *p16* were associated with survival among 105 patients with gastric adenocarcinoma.^68^

## 4. CONCLUSION AND FUTURE DIRECTIONS

Because DNA methylation data are very complex and diverse, several points should be considered in study design and data analysis. For example, data could represent methylation content, methylation level, methylation pattern, methylation level profile, or methylation pattern profile.^69^ Second, the format of data might be discrete (qualitative measurement) or continuous (quantitative measurement), depending on the detection method. When continuous data are not normally distributed, they can be transformed or classified into groups for parametric analysis or, alternatively, tested by nonparametric analysis.^70^ However, methods of statistical analysis of methylation pattern and methylation profile have not been standardized, and the establishment of such techniques should be a topic of future studies.

A well-designed epidemiologic study is needed to evaluate the validity of a putative methylation-based biomarker. Strategies to validate biomarkers were well established in the community of the Early Detection Research Network (EDRN).^71^^,^^72^ The researchers developed a 5-phase strategy for identifying cancer biomarkers. These phases corresponded to 5 epidemiologic phases, as follows: (1) a preclinical exploratory phase in case-control studies with convenient samples, to identify promising directions; (2) a clinical assay and validation phase in population-based case-control studies, for validation in clinical settings; (3) a retrospective longitudinal phase in nested case-control studies, to determine whether the biomarker detects a disease before it becomes clinically significant; (4) a prospective screening phase in cross-sectional cohort studies, to identify the extent and characteristics of a disease and calculate the predictive value^73^; and (5) a cancer control phase in randomized trials, to quantify the impact of screening on reducing the disease burden in the population. The standard operating procedures of each phase should include details of assays, methods, and protocols for collection and processing of biological samples and other reference materials. In addition, with regard to the characteristics of methylation, the timing of environmental exposure may be critical for altering methylation in disease progression; thus, specimen collection should be done in a prospective longitudinal study and should be done repeatedly.

More data must be collected to determine if blood-based DNA methylation is biologically plausible. In addition, a large prospective study is necessary to determine whether DNA methylation measured in peripheral blood is a useful and informative cancer biomarker.
